# Dancing for Health and Wellbeing: A Feasibility Study of Examining Health Impacts of Online Dancing among Pulmonary Fibrosis Patients

**DOI:** 10.3390/ijerph192013510

**Published:** 2022-10-19

**Authors:** Vikram Niranjan, Giampiero Tarantino, Jaspal Kumar, Nicola Cassidy, Liam Galvin, Gemma O’Dowd, Tracey Barnes, Finola O’Neill, Matthew Cullen, Ray O’Connor, Andrew O’Regan

**Affiliations:** 1 School of Public Health, Physiotherapy and Sports Science, University College Dublin, D04 V1W8 Dublin, Ireland; 2 NUS Centre for Cancer Research, Yong Loo Lin School of Medicine, National University of Singapore, Singapore 119077, Singapore; 3 Irish Lung Fibrosis Association, D01 V9Y4 Dublin, Ireland; 4 Dancing for Health CIC, Derbyshire S18 2GR, UK; 5 School of Medicine, University of Limerick, V94 T9PX Limerick, Ireland

**Keywords:** dance, wellbeing, quality of life, community-engaged research, chronic disease, pulmonary fibrosis, pulmonary diseases

## Abstract

Background: Physical activity (PA) is recommended in the management of patients with pulmonary fibrosis (PF) to improve health outcomes. Dance is one such form of PA which is meaningful, valuable, enjoyable and has demonstrated positive physical and mental health effects. Methods: With pre-post design, 16 patients, members of the Irish Lung Fibrosis Association, were enrolled in this study. Once weekly, 75-min dance sessions were delivered for eight weeks via Zoom by an experienced choreographer. Participants completed Chronic Respiratory Questionnaire Self-Administered Standardised Format (CRQ-SAS) and European Quality of Life 5 Dimensions 3 Level Version (EQ-5D-3L) to assess self-rated quality of life. A paired-sample t-test was employed to assess the mean differences between the pre-and post-intervention scores. Results: Most patients (78.57%) were aged over 60 years; with 71.43% diagnosed with pulmonary fibrosis more than 3 years ago. We performed an analysis of 10/16 participants who completed the intervention (5 males, 5 females). On CRQ-SAS scale we found, (a) dyspnoea—small to moderate magnitude improvement of 0.5–1.0 among 50%, (b) fatigue—small to moderate magnitude improvement of 0.5–1.0 among 40%, (c) emotional function—small to high magnitude improvement of 0.5–2.0 among 50%, (d) mastery—small magnitude improvement of 0.5 among 20%. Participants reported their health moderate to best on Visual Analogue Scale of EQ-5D-3L which improved by 1–3 scale among 40%. Mental health improved as percentage of not feeling anxious or depressed rose post event from 42.86% to 72.73%. Conclusion: Our findings demonstrate that a virtual dance intervention is acceptable, enjoyable and feasible for improving health outcomes among PF patients. More organised and continuous events in future may reveal cost-benefit ratio and impact on health outcomes.

## 1. Introduction

Chronic pulmonary diseases (CPD) are among the leading causes of mortality worldwide [1] and it has been estimated that almost 550 million people live with a chronic respiratory disease globally [2]. CPD are usually characterised by high dyspnoea levels, low tolerance for physical activity and exercise and reduced mobility [3], which are leading causes of disability [4]. Moreover, research has highlighted that patients living with CPD are more likely to (i) develop symptoms related to anxiety and depression [5]; (ii) have poorer quality of life compared to healthy people [6]; (iii) have balance-related problems, such as falls [7,8]; and (iv) spend less time engaging in daily physical activity than healthy people [9]. Idiopathic pulmonary fibrosis (IPF) is a rare, chronic, progressive, fibrosing interstitial pneumonia of unknown aetiology and is an incurable disease [10]. The annual incidence in Europe ranges between 0.22 and 7.4 per 100,000 population [11]. The prevalence of IPF in Ireland varies from 3 to 20 cases per 100,000 in the general population [12]. Management of patients with pulmonary diseases, including IPF, include interventions such as daily physical activity [10,11,13,14].

Sufficient levels of physical activity are associated with a reduced risk of hospitalisation and mortality [14,15,16]. In this sense, exercise has been widely acknowledged to be an important tool to improve functional capacity, reduce breathlessness, fatigue and dyspnoea levels [17,18,19,20], and contribute to help people living with CPD being more engaged in daily physical activity [21]. However, despite these benefits and the global guidelines that recommend engaging in at least 30 min a day of moderate physical activity [22], people with CPD still fail to engage in regular physical activity [23]. Pulmonary rehabilitation (PR) as exercise is often delivered to patients at physiotherapy clinics or community settings, which may not be feasible for patients to attend and has higher dropout rates [24]. Research has shown that low motivational levels, the lack of suitable exercise availability and the fear of breathlessness during activities are the most common barriers to exercise among people living with CPD [25].

In this context of physical activity, dance has been highlighted to be a worthwhile and novel form of movement [26], which is beneficial in terms of improved physical performance, mood levels and social interactions among older adults [27]. Furthermore, dance has been also reported to have beneficial effects on people living with neurological and cardiovascular disorders [28,29,30,31]. In relation to people living with CPD, although very limited, previous research has shown that dance activities may improve functional exercise capacity, balance, dyspnoea levels, disease control and emotional function [26,32] and reduce depression levels [32]. Qualitative research has also shown that people living with CPD enjoyed dance sessions and voiced them to be advantageous for their mental and physical health and their social cohesion [33,34].

However, despite dance being acknowledged to be one of the most enjoyable, safe, feasible and low-cost interventions in respiratory care [34,35], there is still limited evidence of the feasibility of dance-based interventions and their potential benefits on people with CPD. One study conducted in this research area reported that the dance intervention was safe and feasible [26]. However, considering the current global pandemic and potential consequences that the exposure to COVID-19 may have on such a vulnerable population, research evidence is absent regarding the feasibility of online dance interventions for people living with CPD. There are no specific research studies available to assess the impact of dancing on the health and wellbeing of pulmonary fibrosis (PF) patients. The Irish Lung Fibrosis Association (ILFA) has delivered online exercise and yoga sessions for patients with PF in Ireland and found that patients prefer using online systems due to the pandemic. Hence, we proposed our research to examine the health impacts of online dancing among pulmonary fibrosis patients in Ireland. The aim of this feasibility study is to evaluate the structured online dancing programme’s physical and mental health benefits among patients with PF in Ireland. The objectives of this study are: 1. To assess the physical and mental health impacts of online dance intervention—(i) quality of life; (ii) dyspnoea levels, fatigue, emotional function and disease control; (iii) anxiety and depression levels; and (iv) health self-perception and 2. To assess the feasibility of dance intervention, we report on (1) the community engagement processes: idea conception and information about a structured online dance programme for individuals living with pulmonary fibrosis in Republic of Ireland; (2) methods and findings: delivery of dance programme and information reported from pre-post intervention measured via self-reported questionnaires; (3) feasibility aspects: acceptability, response and adherence. This information will be crucial for future planning of a sustainable community-based dance programme as a large-scale definitive research trial.

## 2. Methods

### 2.1. Community Engagement/Patient and Public Involvement (PPI) Processes

The ILFA is the national patient organisation that supports patients and families living with PF and provides education and support to respiratory healthcare professionals. ILFA is associated with various community development projects for patients with pulmonary fibrosis and their carers. A survey of patients engaged in online exercise classes revealed that patients would like to have a dance intervention. VN (Lead researcher) approached and discussed the online dance intervention with the ILFA. The ILFA played a key role as co-authors in preparing for funding application, helping to establish the communication between researcher and the patients. They also helped by planning and delivery of the programme, providing feedback on the questionnaires and evaluation reports and actively participating in research dissemination activity. The ILFA organised a social online interaction event entitled ‘Let’s talk dance’. This took place on 5 April 2022 with the aim to establish communication between researcher, choreographer (TB) and the patients. At this event, the dance intervention programme was explained, and queries of the patients and their carers were addressed. TB shared videos of previous dance programmes held in the UK as a part of the research and real experiences of those who participated. Our event resulted in on the spot registration by 13 patients. Two patient advisory members of the ILFA (FON and MC), who also participated in the study, were enthusiastically involved in the writing and reviewing of the manuscript.

### 2.2. Research Design

Our study adopted a transdisciplinary approach to measure the health and wellbeing impact and feasibility aspects via pre-post intervention testing [36]. An equal status, simultaneous mixed methods study design was applied. This paper reports the quantitative analysis of the study.

Ethical approval for this study was granted by the institutional Human Research Ethics Committee—Sciences, UCD with reference No. LS-21-94-Niranjan.

### 2.3. Participants and Setting

A group of 16 patients with PF, residing in Ireland and of age group >25 years was recruited after the ‘Let’s talk dance’ event. At the initial self-assessment screening, patients confirmed the diagnosis of PF and that they were in a stable clinical state at the time of the study. We used Zoom for delivering online dance sessions. Patients participated from their home and informed their family regarding participation in the activity. Patients were advised to attend the dance sessions in the presence of a family member or carer. Participants were given full programme information with safety instructions and upon reading, signed an informed consent before taking part in the study.

### 2.4. Dance Programme

Every Tuesday, live dance classes, of seventy-five minutes duration, were offered for eight consecutive weeks. They were delivered by TB online; GT observed the participants for any adverse condition for all dance sessions and VN attended two sessions to offer motivation. TB danced with the group, demonstrating the dances in advance where necessary, but otherwise inviting participants to follow along.

The seated dance programme primarily focused on arm movements to music. Each routine was specifically choreographed to the music track used. In developing each of the seated dance routines the choreography included either, intro, verse and chorus choreography or was structured using the 32 beat phrases or beats in the music. Actions to words in the songs were also used, making the routines fun and easy to learn.

The music selection was from various genres, from the 1950s up to present day tracks. The music selection included familiar and recognisable songs, with artists like, Gene Kelly, Elvis, Abba, Neil Diamond, Diana Ross, Ed Sheeran and Justin Timberlake. The tracks selected had either a slow or a more upbeat tempo. Slower tempo tracks were used for the warmup, cool down and stretches.

Each class started with a 5-min warm up, as to mobilise the muscles and joints in the arms and upper body and prepare for the class. Each time the choreographer demonstrated the dance moves for a couple of minutes, followed by a song. Each song ran for 5 to 6 min, during which all the participants danced along with the choreographer. Through the main section of the dance class, a faster tempo track was alternated with a slower tempo track. A talk through and demonstration before each track allowed the participants a little pause and rest between songs.

The class finished off with a 5-min cool down. These slower movements lowered the pulse and stretched out the major muscles that were used in the dancing session. The participants selected some of their favourite Irish songs which were used when doing the warmups and cool downs. Having upbeat and uplifting music helped elevate the mood as well as engage and motivate the participants through each section of the class.

### 2.5. Data Collection

All the participants were asked to complete self-assessment questionnaires. It comprised of demographic characteristics of participants, their experience and satisfaction with and recommendation for the dance programme and two measures of self-rated quality of life. The Chronic Respiratory Questionnaire Self-Administered Standardised Format (CRQ-SAS) has been developed for use as a measure of quality of life for patients with chronic pulmonary disease [37]. It records patients’ experiences and feelings in the last two weeks under four domains: dyspnoea, fatigue, emotional function and mastery. The European Quality of Life 5 Dimensions 3 Level Version (EQ-5D-3L) questionnaire was used for quality-of-life analysis, which is a descriptive system, comprising of the following five dimensions: mobility, self-care, usual activities, pain/discomfort and anxiety/depression [38]. Each dimension has three levels: no problems, some problems and extreme problems. The participant was asked to indicate his/her health state by ticking the box next to the most appropriate statement in each of the five dimensions. The EQ Visual analogue scale (VAS) is the second part of the questionnaire, asking to mark health status on the day of the interview on a 20 cm vertical scale with endpoints of 0 and 100. VAS records the patient’s self-rated health on a vertical visual analogue scale where the endpoints are labelled ‘Best imaginable health state’ and ‘Worst imaginable health state’. The VAS can be used as a quantitative measure of health outcome that reflects the patient’s judgement.

### 2.6. Data Analysis

Data were encrypted, anonymised and stored in password protected file with access to PI (VN) only. We did comparative analysis with pre and post intervention data using SPSS version 27 (IBM Corp., Armonk, NY, USA). A paired-sample *t*-test was employed to assess the mean differences between the pre-and post-intervention scores. The effect size of the mean difference was assessed using Cohen’s *d* (the difference between the pre- and post-intervention scores divided by the pooled standard deviation). The following scale of magnitude was used to assess the effect sizes: <0.19 = trivial; 0.20 to 0.49 = small; 0.50 to 0.79 = moderate; and ≥0.80 = large [39]. However, following Hopkins’ guidelines [40], the uncertainty in the estimate or effect in this study were interpreted in terms of their magnitude and respective upper and lower confidence limits.

However, we also cross validated the magnitude of the mean differences following the CRQ-SAS guidelines, which suggest that when a respondent’s score improves an average of 0.5 per question per dimension, respondents generally report that they have been feeling better, and that the magnitude of change, while small, is important to their day-to-day lives. Changes between 0.75 and 1.25 represent important changes of moderate magnitude, and changes greater than 1.5 represent important changes of large magnitude [41,42].

## 3. Results

### 3.1. Participants’ Characteristics

Twenty members of the Irish Lung Fibrosis Association (ILFA) were contacted to participate in this study, of which 16 agreed to attend the dance sessions. At the baseline, the majority of the participants (80%) were older than 60 years, whereas 62.5% were females (*n* = 10). In terms of pulmonary conditions, 64.3% of the participants (*n* = 9) had IPF, whereas the remaining participants had other pulmonary conditions (e.g., systemic sclerosis, hypersensitivity pneumonitis and interstitial pulmonary disease due to antecedent fibrosis). Finally, all the participants had previously attended an online physical activity programme. The demographic characteristics of the study participants are fully reported in Table 1.

### 3.2. Feasibility of the Intervention

Of the twenty members of the ILFA approached, only 16 were interested in the programme (80%), of which 10 completed the programme (completion rate = 62.5%). Of the 16 participants that expressed their interest to attend the programme, three withdrew from the intervention after completing the baseline questionnaires and attending the first session with no reasons given, and a further three participants did not complete the intervention due to a number of different reasons (pre-existing shoulder and back pain, not interested in dance activities and holiday already planned).

The average number of participants during the dance sessions was 9 ± 4, with a minimum of 5 participants and a maximum of 16 participants. The average attendance rate for the participants ranged from 25% (2 sessions) to 100% (8 sessions), with an average value of 51.5% (namely, on average, the participants attended 4 sessions).

There were no adverse effects reported by the participants during the sessions. However, some participants reported shortness of breath while performing the dance activity, which was resolved by taking a short rest during the session. Moreover, despite the dance steps being developed for sitting dance activities, a few (*n* = 2) participants felt comfortable enough to dance while standing.

The responses to the ten statements related to the feasibility, acceptability, satisfaction and recommendation of the online dance intervention were accompanied by a Likert scale ranging from 1 = strongly agree to 5 = strongly disagree and are reported in Table 2 Overall, participants indicated an absence of technical issues, reporting that they could clearly see and hear the dance teacher. All the participants agreed that the programme not only met the expectations (m = 1.82 ± 1.17), but it provided an experience as good as the face-to-face programmes that they had previously attended (m = 2.18 ± 1.08). Finally, participants indicated that they would attend such an online dance programme in the future (m = 2.00 ± 1.00), suggesting acceptability and strongly recommend it to other people living with pulmonary conditions (m = 1.55 ± 0.69).

### 3.3. Estimation of CRQ-SAS

The magnitude of the difference in the CRQ-SAS domains are reported in Table 3. Noteworthy is the extent to which the fatigue levels slightly improved after the intervention. Despite the unclear differences that our results revealed (mean difference = 0.13; *d* = 0.17; CIs = −0.462 to 0.787), participants reported that they felt less tired during the two weeks prior to post-intervention questionnaire. Furthermore, despite unclear, small-sized negative differences being found in terms of the disease control (mean difference = −0.10; *d* = −0.23; CIs = −0.856 to 0.401), with participants reported that they felt less fearful when having difficulties to breathe (pre-intervention mean = 6.20; post-intervention mean = 6.30) and more confident about dealing with the illness (pre-intervention mean = 2.80; post-intervention mean = 2.70).

In terms of gender differences, our findings revealed that females had better emotional function than males, both pre- and post-intervention (Table 4). In fact, our analyses revealed possible trivial-to-large-sized differences between males and females regarding the emotional function after participating in the dance sessions (mean difference = −0.62; *d* = −1.25; CIs = −2.622 to 0.181), with females reporting that they felt less frustrated, upset, discouraged and restless and more relaxed and satisfied with their life than males. These differences were also found before the intervention, with the analyses revealing small-sized, although unclear, differences between males and females (mean difference = −0.22; *d* = −0.46; CIs = −1.733 to 0.837).

### 3.4. Estimation of EQ-5DL

The magnitude of the differences in the quality-of-life questionnaire are reported in Table 5. Despite the small sample size and the unclear effects, our investigation revealed small-sized differences in the anxiety levels among the participants, which slightly decreased after the intervention (mean difference = −0.11; *d* = −0.33; CIs = −0.996 to 0.349). Small-sized, although unclear, differences were also found in the mobility capability (mean difference = 0.11; *d* = 0.33; CIs = −0.349 to 0.996), which increased after participating in the dance sessions. Finally, unclear small-sized differences were found in the health self-perception (mean difference = 0.40; *d* = 0.23; CIs = −0.401 to 0.856), which increased after participants attended the 8-week dance intervention.

In terms of gender differences, Table 6 summarises the mean differences between gender. Noteworthy is the extent to which females had lower anxiety levels both before and after the dance sessions. Our analyses revealed unclear large-sized differences before the dance sessions (mean difference = 0.55; *d* = 1.17; CIs = −0.310 to 2.581) and moderate-to-large-sized differences after participation in the intervention (mean difference = 0.75; *d* = 2.45; CIs = 0.681 to 4.140).

## 4. Discussion

### 4.1. Summary

To the authors’ knowledge, this is the first study to explore the use of an online dance-based exercise intervention in the PF population. Our feasibility study showed that the 8-week online dance programme was successfully delivered to patients with PF and found it to be safe, enjoyable, beneficial and feasible. The online and seated mode was acceptable to participants with a wide range of ages and disease severity.

There was substantial engagement between dance choreographer, research member and participants, as shown in Table 2. The Likert scale results indicate high levels of acceptability and technical feasibility and is evidence of the potential for this population fully engaging with a future intervention. The results are promising in the context of a population that may have challenges attending centres for in-person programmes, and at the same time who would not be expected to be familiar with this type of technology. There were no adverse events reported throughout the 8 weeks. Participants found dance classes enjoyable and readily adopted the dance programme.

### 4.2. Comparison to Existing Literature

#### 4.2.1. Acceptance of Dance Programme

The participants in this study showed favourable acceptance towards online dance programme which is similar to the limited existing research regarding dance for people with chronic pulmonary diseases [26,34]. The studies assessing dance intervention among Chronic Obstructive Pulmonary Disease (COPD) patients in Canada and CPD in the UK found it feasible and acceptable. Moreover, both studies reported improvements in functional capacity, balance, anxiety and depression, physical activity and health-related quality of life. The friendly, engaging group, safe and home-comfort environment of the dance program could serve as a motivating factor as opposed to exercising alone. Our study participants found that their mood has improved, and classes were enjoyable. They also reported that they would like to participate in dance programmes in future. Our 62.5% completion rate indicated high satisfaction of the participants which was encouraging. Our adherence rate is 62.5%, which is higher compared to previously reported 49% in the study conducted by Wshah et al. [26], lower compared to 70% in the study conducted by Harrison et al. [32], 66.66% in the study conducted by Goodill [43] and 80% in the study conducted by Gardiner et al. [44] involving patients with pulmonary diseases. Most of the population in our study were elderly people whose uptake and attendance rate were similar to other studies involving physical activity intervention among older age groups [45,46,47]. In this sense, a systematic review showed that adherence rates were generally higher in supervised programmes [48]. We ensured safety aspects that our participants have someone with them during dance sessions and also one member of our research team was observing all the participants while dancing.

#### 4.2.2. Patient Satisfaction

From a previous survey by ILFA, patients mentioned they might want to try dance while they were attending physiotherapy rehabilitation. The principles of health behavioural change suggest that a choice of exercise styles may assist in improving uptake and adherence to regular physical activity [49]. We heard and responded to the patient’s voice for the dance programme, and that could be the major factor for high level of satisfaction in our study. Feeling valued and the opportunity to suggest music of their own choice could be other factors for high patient satisfaction.

This study identified high levels of satisfaction with the use of technology. This might be due to uninterrupted and good internet and familiarity of the participants to use online platforms post-pandemic. This was similar to another pilot study conducted by Holland et al. [50] in rural Australia where they mentioned that home-based telerehabilitation using readily available equipment is safe and feasible for people with COPD and it requires an adequate data network.

#### 4.2.3. Impact on Health and Wellbeing

Several studies have shown PA can have positive physical and mental health impacts, such as reduced fatigue, breathing efficiency, decreased anxiety, depression, reduced negative psychological effects of confinement and improved mental health [51,52]. A recent systematic review reported a statistically significant association between high levels of PA and lower levels of anxiety and depression, lower levels of insomnia, negative moods and negative emotions [53]. Although trivial to moderate, our feasibility study of dance found similar health improvements particularly decreased fatigue and improved emotional function. Harrison et al. [32] reported that improvement in mental health could be due to functional gains obtained through participation in choreographed dances (e.g., remembering choreography while moving to the beat), which is also the case in our study. Another important outcome, wellbeing aspects, measured with self-reported health perception increased between both males and females in our study. Our study is based on a weekly 75-min dance session, which was similar to other studies [32,33,34]. However, the dose of this dance interventions varied individually within each patient’s ability to actively participate. This ability to participate was related to their level of disability resulting from their pulmonary disease, and the reduced level of physical fitness, which is associated with chronic pulmonary disease.

#### 4.2.4. Practical Considerations

This was a seated dancing intervention which was held online and did not require a partner to participate. There was no requirement of a special dance hall, transport which prevented any cross-infection, especially during COVID-19 times. Singing, dancing and other forms of exercise are prone to aerosol generation and when performed in a group, could be risky for a vulnerable group of patients. Patients with pulmonary disease often have the additional burden of carrying oxygen cylinders with them. Our online, seated, home-based dance intervention reduced the stress associated with the above factors.

#### 4.2.5. Strengths and Limitations of the Study

Being the first study of dance intervention among the PF population, no adverse events, high adherence rate, strong community engagement/PPI and mild to moderate health improvements reported by participants are some of the major strengths of our study. Our calculation of adherence rate was based on the drop-out rate (37.5%) and attendance rate (51.5%). Out of 13 participants, 10 participants completed the programme. Of these ten, 5% attended all eight classes and 70% attended six classes. This was in comparison to the three people who dropped out, all of whom attended only two classes. In terms of adherence to community-based group exercise interventions for older people, one systematic review mentioned that approximately half of participants who commence an exercise programme will drop out within the first six months [54]. Another systematic review indicates that the proportion of older adults completing group exercise programs ranged from 65% to 86%. Moreover, the proportion of sessions attended fluctuated from 58% to 77%, and the average number of home exercise sessions completed per week ranged from 1.5 to 3 [55]. When compared to this evidence, our study shows acceptable level of adherence to the programme.

It was ensured that the dance programme incorporated the needs of the group, their stories, music choices and movement needs. There was collaboration with a community organisation, the ILFA who helped to manage better communication between the patients and the research team. Our ‘Let’s talk dance’ session was an icebreaker where most participants had their queries resolved and this single session helped us to build a good rapport throughout the intervention and a better engagement with the participants. A qualitative study about identifying solutions to increase participation in physical activity interventions mentioned that participants tend to have a meaningful engagement if they experience good awareness of interventions in which they were involved directly, better communication and clarity in planning and organisation of the class [56]. This was very well reflected in our study because of the strong community engagement and PPI. In terms of limitations, first, as this is a feasibility study, there were no statistically significant health and wellbeing improvements reported among the participants for any of the primary or secondary outcome measures in this pre-post intervention study, which may have been due to inadequate power resultant from the small number of programme participants. Second, we could not implement any clinical parameters to measure the physical health impacts such as the 6-min walk test. Third, we only included a specific group of population and there was no control group. So, there is limited generalisability of the findings to other European countries or different parts of the world.

#### 4.2.6. Implications of the Study

At societal and community levels, our study forms a good example to add to the existing evidence and promote good collaboration practices among patients, patient organisations, art performers and academic researchers to enhance civic society. To the limited dance-based research interventions, our evaluation can promote dance as an art form for community engagement and as a valid exercise tool, with potential implementation on a larger scale. This may encourage future intergenerational dance as a physical activity programme.

Health Service Executive (HSE) in Ireland promotes ‘Social prescribing’ which is a means of enabling GPs, nurses and other health care professionals to refer people to a range of non-clinical community supports which may have significant benefits for their overall health and wellbeing [57]. Our study clearly has implications for social prescribing opportunities: it dispels myths that patients with chronic pulmonary diseases having breathlessness cannot participate in dance; and that, they can and should enjoy an activity of their choice and they should have a choice.

At clinical and public health levels, dance can be an alternate exercise-based rehabilitation programme which can be accepted favourably by patients leading to higher adherence rate and better health and wellbeing outcomes. It may also help to achieve higher targets of recommended PA levels as only 30–33% achieve target PA levels in the community according to Ireland Physical Activity Fact sheet [58,59]. A recent systematic review measuring the impact of dance interventions among patients with chronic pulmonary diseases mentioned that dance classes had positive and significant benefits on walking distance, balance and dyspnoea levels as well as quality of life and emotional function. It also found that dance was an enjoyable activity for the participants, allowing them to socialise and overturn the negative effects of physical inactivity [60]. Another systematic review concluded that counselling to promote physical activity in primary care has a limited effect on patients’ behaviour and it might not, on its own, be enough to change physical activity behaviour [61]. As a consequence, this feasibility study should progress to a fully powered RCT because: (a) More than 40% of participants provided reliable data for daily determination of time spent in moderate to vigorous exercise, (b) more than 40% of participants maintained engagement with the intervention and (c) potential for behaviour change motivation established by the intervention showed potential to fully achieve the desired outcomes. A full trial, adequately powered for outcome calculations, and involving a dance intervention of longer duration, as well the inclusion of clinical parameters in data collection, would potentially add meaningful and new data to this under researched area.

## 5. Conclusions

Our findings demonstrate that a virtual dance intervention is acceptable, enjoyable and feasible for improving health outcomes among PF patients in Ireland. As dance is a low-cost activity that can be done at home, dance interventions may be used as an exercise pathway for patients with pulmonary diseases. Standard dance intensity, frequency and duration based on age and pulmonary efficiency level suitability would encourage the participants. Our transdisciplinary research approach and engagement with community organisation were paramount to establishing the intervention, exploring its benefits and ensuring its sustainability. More organised and continuous events in future may reveal cost-benefit ratio and impact on health outcomes.

## Figures and Tables

**Table 1:** Baseline participants’ demographic characteristics.

Variable	*n* (%)
Gender (*n* = 16)	
Females	10 (62.5)
Males	6 (37.5)
Age (*n* = 15)	
31–39	1 (6.7)
40–50	0 (0.0)
51–60	2 (13.3)
61–70	5 (33.3)
71–80	4 (26.7)
81–90	3 (20.0)
Diagnosis (*n* = 14)	
Idiopathic Pulmonary Fibrosis	9 (64.3)
Other	5 (35.7)
How long ago have you been diagnosed? (*n* = 15)	
Less than 6 months	1 (6.7)
More than 6 months and less than 1 year	1 (6.7)
More than 1 year and less than 3 years	2 (13.3)
More than 3 years	11 (73.3)
Are you currently: (*n* = 15)	
Employed	1 (6.7)
Retired	11 (73.3)
Unable to work due to my condition	3 (20.0)
Student	0 (0.0)
Due to COVID-19, are you currently: (*n* = 15)	
Always staying indoors	1 (6.7)
Sometimes going out using face covering	9 (60.0)
Sometimes going out without face covering	5 (33.3)
Have you participated in online physical activity programmes before? (*n* = 15)	
Yes	15 (100.0)
No	0 (0.0)

**Table 2:** Feasibility, acceptability, satisfaction and recommendation.

Question	Mean (sd)
Were there technical problems? (*n* = 11)	4.36 (0.81)
Were you comfortable attending the online class? (*n* = 10)	1.90 (1.20)
Were you comfortable with your privacy? (*n* = 10)	1.50 (0.53)
Were you able to clearly hear the teacher? (*n* = 11)	1.36 (0.51)
Were you able to clearly see the teacher? (*n* = 11)	1.73 (1.20)
Were you given clear information beforehand attending the online programme? (*n* = 10)	1.30 (0.48)
Did the online dance programme meet your expectations? (*n* = 11)	1.82 (1.17)
Does the online dance class provide as good an experience as face-to-face? (*n* = 11)	2.18 (1.08)
Will you participate again in the online dance programme in the future? (*n* = 11)	2.00 (1.00)
Would you recommend the online dance programme to others? (*n* = 11)	1.55 (0.69)

Likert scale accompanying the questions: 1 = “Strongly Agree” 5 = “Strongly Disagree”. sd, standard deviation.

**Table 3:** Mean differences of the Chronic Respiratory Questionnaire Self-Administered Standardised Format (CRQ-SAS) domains.

Domains (*n* = 10)	Before	After	Mean Difference	Cohen’s *d*	Lower CI	Upper CI	*p* Value
Dyspnoea	5.48	5.50	0.02	0.02	−0.598	0.642	0.944
Fatigue	4.05	4.18	0.13	0.17	−0.462	0.787	0.610
Emotional Function	4.67	4.70	0.03	0.06	−0.563	0.677	0.857
Disease Control	4.43	4.33	−0.10	−0.23	−0.856	0.401	0.479

Likert scale accompanying the questions: 1 = all the time; 7 = none of the time. CI, confidence interval. Changes between 0.75 and 1.25 represent important changes of moderate magnitude and changes greater than 1.5 represent important changes of large magnitude.

**Table 4:** Chronic Respiratory Questionnaire Self-Administered Standardised Format (CRQ-SAS) Gender differences.

Domain (*n* = 10)	Mean	Cohen’s *d*	Lower CI	Upper CI	*p* Value
Dyspnoea					
Pre-intervention					
Males	5.55	0.11	−1.164	1.368	0.874 ^a^
Females	5.43				
Post-intervention					
Males	5.50	0.00	−1.265	1.265	1.000 ^a^
Females	5.50				
Fatigue					
Pre-intervention					
Males	4.13	0.14	−1.128	1.406	0.830 ^a^
Females	4.00				
Post-intervention					
Males	4.19	0.03	−1.235	1.295	0.963 ^a^
Females	4.17				
Emotional Function					
Pre-intervention					
Males	4.54	−0.46	−1.733	0.837	0.495 ^a^
Females	4.76				
Post-intervention					
Males	4.33	−1.25	−2.622	0.181	0.088 ^a^
Females	4.95				
Mastery					
Pre-intervention					
Males	4.56	0.38	−0.908	1.649	0.570 ^a^
Females	4.33				
Post-intervention					
Males	4.31	−0.03	−1.291	1.240	0.968 ^a^
Females	4.33				

^a^: equal variances assumed. CI, confidence interval. Likert scale accompanying the questions: 1 = all the time; 7 = none of the time. Changes between 0.75 and 1.25 represent important changes of moderate magnitude and changes greater than 1.5 represent important changes of large magnitude.

**Table 5:** Mean differences of the European Quality of Life 5 Dimensions 3 Level Version (EQ-5D-3L) items.

Items	Before	After	Mean Difference	Cohen’s *d*	Lower CI	Upper CI	*p* Value
Mobility	1.22	1.33	0.11	0.33	−0.349	0.996	0.347
Self-Care	1.10	1.30	0.20	0.47	−0.194	1.119	0.168
Usual Activities	1.60	1.60	0.00	0.00	−0.620	0.620	1.000
Pain/Discomfort	1.30	1.30	0.00	0.00	N/A	N/A	1.000
Anxious	1.44	1.33	−0.11	−0.33	−0.996	0.349	0.347
Health Perception	7.50	7.90	0.40	0.23	−0.401	0.856	0.479

Likert scale accompanying the questions: 1 = “I have no problem with…”; 2 = “I have some problem with…”; 3 = “I have problem with…”. Health Self-perception: 0 = “extremely bad”; 10 = “extremely good”. CI, confidence interval, N/A: Non attainable.

**Table 6:** European Quality of Life 5 Dimensions 3 Level Version (EQ-5D-3L) Gender differences.

Item (*n* = 10)	Mean	Cohen’s *d*	Lower CI	Upper CI	*p* Value
Mobility					
Pre-intervention					
Males	1.25	0.19	−1.087	1.450	0.779 ^a^
Females	1.17				
Post-intervention					
Males	1.33	0.00	−1.386	1.386	1.000 ^a^
Females	1.33				
Self-Care					
Pre-intervention					
Males	1.00	−0.52	−1.791	0.788	0.447 ^a^
Females	1.17				
Post-intervention					
Males	1.00	−0.76	−2.051	0.582	0.203 ^b^
Females	1.50				
Usual Activities					
Pre-intervention					
Males	1.50	−0.23	−1.489	1.050	0.735 ^a^
Females	1.67				
Post-intervention					
Males	1.50	−0.31	−1.573	0.975	0.645 ^a^
Females	1.67				
Pain/Discomfort					
Pre-intervention					
Males	1.25	−0.16	−1.426	1.109	0.807 ^a^
Females	1.33				
Post-intervention					
Males	1.25	−0.16	−1.426	1.109	0.807 ^a^
Females	1.33				
Anxiety/Depression					
Pre-intervention					
Males	1.75	1.17	−0.310	2.581	0.125 ^a^
Females	1.20				
Post-intervention					
Males	1.75	2.45	0.681	4.140	0.058 ^b^
Females	1.00				
Health Self-Perception					
Pre-intervention					
Males	7.75	0.24	−1.038	1.503	0.720 ^a^
Females	7.33				
Post-intervention					
Males	8.00	0.12	−1.148	1.385	0.854 ^a^
Females	7.83				

^a^: equal variances assumed. ^b^: equal variances not assumed. Likert scale accompanying the questions: 1 = “I have no problem with…”; 2 = “I have some problem with…”; 3 = “I have problems with…”. Health Self-perception: 0 = “extremely bad”; 10 = “extremely good”. CI, confidence interval.

## Data Availability

The data presented in this study are available on request from the corresponding author. The data are not publicly available due to ethical reasons. Written informed consent has been obtained from the patient(s) to publish this paper.
